# *γδ*T Cells Exacerbate Podocyte Injury via the CD28/B7-1-Phosphor-SRC Kinase Pathway

**DOI:** 10.1155/2018/5647120

**Published:** 2018-05-15

**Authors:** Wanbing Chen, You Wu, Gaofu Zhang, Mo Wang, Haiping Yang, Qiu Li

**Affiliations:** ^1^Key Laboratory of the Ministry of Education, Key Laboratory of Pediatrics in Chongqing, Chongqing International Science and Technology Cooperation Center for Child Development and Disorders, Chongqing Key Laboratory of Child Infection and Immunity, Children's Hospital of Chongqing Medical University, Chongqing, China; ^2^Children's Hospital of Chongqing Medical University, No. 136 Second Zhongshan Road, Yuzhong District, Chongqing 400014, China; ^3^Department of Nephrology, Children's Hospital of Chongqing Medical University, Chongqing, China

## Abstract

Primary nephrotic syndrome (PNS) is a devastating pediatric disorder. However, its mechanism remains unclear. Previous studies detected B7-1 in podocytes; meanwhile, *γδ*T cells play pivotal roles in immune diseases. Therefore, this study aimed to assess whether and how *γδ*T cells impact podocytes via the CD28/B7-1 pathway. WT and TCR*δ*^−/−^ mice were assessed. LPS was used to induce nephropathy. Total *γδ*T and CD28^+^*γδ*T cells were quantitated in mouse spleen and kidney samples. B7-1 and phosphor-SRC levels in the kidney were detected as well. In vitro, *γδ*T cells from the mouse spleen were cocultured with mouse podocytes, and apoptosis rate and phosphor-SRC expression in podocytes were assessed. Compared with control mice, WT mice with LPS nephropathy showed increased amounts of *γδ*T cells in the kidney. Kidney injury was alleviated in TCR*δ*^−/−^ mice. Meanwhile, B7-1 and phosphor-SRC levels were increased in the kidney from WT mice with LPS nephropathy. CD28^+^*γδ*T cells were decreased, indicating CD28 may play a role in LPS nephropathy. Immunofluorescence colocalization analysis revealed a tight association of *γδ*T cells with B7-1 in the kidney. High B7-1 expression was detected in podocytes treated with LPS. Podocytes cocultured with *γδ*T cells showed higher phosphor-SRC and apoptosis rate than other cell groups. Furthermore, CD28/B7-1 blockage with CTLA4-Ig in vitro relieved podocyte injury. *γδ*T cells exacerbate podocyte injury via CD28/B7-1 signaling, with downstream involvement of phosphor-SRC. The CD28/B7-1 blocker CTLA4-Ig prevented progressive podocyte injury, providing a potential therapeutic tool for PNS.

## 1. Introduction

Primary nephrotic syndrome (PNS), characterized by proteinuria, hypoalbuminemia, hyperlipidemia, and edema, has become a devastating disorder in children. PNS can be divided into minimal change disease (MCD), focal segmental glomerulosclerosis (FSGS), mesangial proliferative glomerulonephritis (MPGN), and membranous nephropathy (MGN). Among them, MCD accounts for nearly 80% of pediatric cases. Most children with MCD respond well to corticosteroid treatment, but relapse and corticosteroid resistance still constitute important clinical challenges. Nevertheless, lack of knowledge regarding PNS mechanism is a barrier in developing efficient therapeutic methods.

In 1974, Shalhoub proposed that T cell dysfunction could be the mechanism underlying nephrosis [1]. Since then, subsets of altered CD4^+^T cells have been reported in PNS patients. We also found Th17/Treg subset dysfunction in pediatric PNS [2]. However, this phenomenon cannot totally explain the mechanism of PNS, especially in its early phase. Therefore, more studies are required.

According to TCR peptide chains, T cells can be divided into* αβ*T and* γδ*T cells. Different from* αβ*T cells,* γδ*T cells are not subject to MHC restriction, which results in* γδ*T cells being activated prior to* αβ*T cells [3]. Therefore,* γδ*T cells are considered innate-like immune cells and play a crucial role in the immunologic balance and early pathological process of diseases such as pancreatic oncogenesis, autoimmune arthritis, and crescentic glomerular nephropathy [4–6]. Our study was aimed to study whether* γδ*T cells could participate in PNS.

Podocytes are among the most important members of the glomerular infiltration barrier. Previous findings confirmed podocyte injury is one of the leading causes of MCD. However, several studies demonstrated that podocytes can be regarded as immune-like cells because of B7-1 expression. Furthermore, elevated B7-1 expression on podocytes is positively correlated with podocyte injury [7–12]. B7-1, also termed CD80, is normally expressed on the surface of antigen processing cells. As a costimulatory molecule, B7-1 can bind CD28 on T cells, providing stimulatory signals for both cells. Based on the immunological characteristics of podocytes and* γδ*T cells, we sought to assess whether and how their interactions through CD28-B7-1 signaling could affect the structure and function of podocytes.

SRC is a nonreceptor tyrosine kinase family member that modulates several cellular events. Buvall and colleagues confirmed that phosphor-SRC activation could lead to synaptopodin (a podocyte's cytoskeleton proteins) dephosphorylation, ultimately resulting in loss of stress fibers and podocyte injury [13]. Combining the finding that CD28-B7-1 binding could activate intracellular SRC kinase [14], we hypothesized that* γδ*T can provide activation signals for SRC kinase which leads to synaptopodin dephosphorylation through CD28/B7-1, causing podocyte injury ultimately. The present study assessed the CD28/B7-1-pSRC pathway in* γδ*T cells and podocytes both in vitro and in vivo.

## 2. Materials and Methods

### 2.1. Mice and Podocytes

Thirty-five eight-week-old female C57BL/6 WT mice were divided into five groups, including the control and 4 (1, 3, 5, and 7 days) LPS nephropathy groups. Meanwhile, 10 eight-week-old female B6.129P2-TCR*δ*tm/Mom/J (TCR*δ*^−/−^ for short) mice on the C57BL/6 background were kindly provided by Professor Zhinan Yin at Jinan University through Professor Xiaodong Zhao at Children's Hospital of Chongqing Medical University. All mice were housed under specific pathogen-free conditions. The animals were injected intraperitoneally with either 200 *μ*g LPS (Sigma,* E. coli* 0111:B4. 1 mg/ml in sterile LPS-free PBS) sterile LPS-free PBS, in equal volumes of 200 *μ*l [9, 15, 16]. Twenty-four-hour urine samples were collected after LPS treatment at 0, 1, 2, 3, 4, 5, 6, and 7 days, respectively. Mice were sacrificed at 0, 1, 3, 5, and 7 days, respectively, and organs were collected. The experimental protocol was approved by the Animal Care Committee of Chongqing Medical University.

Conditional immortalized mouse podocytes were a gift from Professor P. Mundel of Mount Sinai School of Medicine through Professor Zhuo Yang at Nankai University and cultured as described elsewhere [17]. Podocytes were divided into five groups, including Groups A, B, C, D, and E, representing the control group, LPS treatment group without* γδ*T cells, LPS treatment group cultured with* γδ*T cells in the upper well of the transwell plate (0.4 *μ*m, Corning), LPS treatment group cocultured with* γδ*T cells, and LPS treatment group cocultured with* γδ*T cells and treated with CTLA4-Ig (Novoprotein, 100 *μ*g/ml), respectively. Podocytes were exposed to LPS (50 *μ*g/ml, 100 *μ*g/ml) for treatment [9].

### 2.2. Urine Protein/Urine Creatinine Determination

Urine protein was detected by the Coomassie Brilliant Blue method (Tiangen). Urine (1 *μ*l), ddH2O (14 *μ*l), and CBB staining solution (285 *μ*l) were added to 96-well plates. Protein concentration was assessed on Thermo Scientific Microplate Reader at 595 nm. Urine creatinine was detected with Creatinine Assay Kit (Nanjing Jiancheng Bioengineering Institute), according to the manufacturer's instructions. Finally, urine protein was normalized to urine creatinine.

### 2.3. Light and Electron Microscopy

For light microscopy, mouse kidneys were perfused with PBS via the left cardiac ventricle and fixed with 4% paraformaldehyde for at least 24 hours. Then, mouse kidneys were paraffin embedded, cut into 3 mm thick kidney sections, stained with hematoxylin and eosin, and finally observed by light microscopy.

For electron microscopy, fresh mouse kidneys were sliced into 1 mm3 sections and fixed with 2.5% glutaraldehyde in 0.1 M cacodylate buffer for 1 hour at pH 7.4, washed in the same buffer, postfixed by incubation in 1% OsO4 for 45 min, and placed in 0.5% aqueous uranyl acetate for 1 hour at 4°C. Tissues were next dehydrated in graded ethanol, infiltrated with a mixture of propylene oxide and Epon resin, and embedded in Epon. Ultra-thin sections were obtained on an EM UC6 ultramicrotome (Leica) and stained with toluidine blue for glomerulus selection. Ultrathin sections were obtained and poststained with uranyl acetate and lead citrate and examined under a Philips Tecnai 12 electron microscope.

### 2.4. Flow Cytometry

Spleen and kidney specimens were obtained from the mice after euthanasia, homogenized, and filtered through 70 *μ*m cell strainers (BD Falcon) for single-cell suspension preparation. Red blood cells were lysed three times with ammonium chloride-potassium lysing buffer (Quality Biological, Inc.) for 5 min. The obtained cells were counted with a cell counter (Countstar) and adjusted to 1*∗*105/ml. The cells were transferred into flow cytometry tubes and stained with anti-mouse gamma delta TCR FITC or Armenian hamster IgG isotype control FITC (eBioscience) for 30 min. This was followed by staining with anti-mouse CD3e PE-Cyanine5.5 (eBioscience), PE/cy7 anti-mouse CD28 or PE/cy7 mouse IgG2b, *Κ* isotype ctrl (BioLegend), and APC anti-mouse CD195 (CCR5) or APC Rat IgG2a, *Κ* isotype ctrl (BioLegend), and Brilliant Violet 421 anti-mouse CD192 (CCR2) antibody or Brilliant Violet 421 Rat IgG2b, *Κ*isotype ctrl (BioLegend) for 30 min at 4°C in the dark. Flow cytometry was performed on a FACSCanto II (BD); data analysis was carried out with the FlowJo software (Tree Star).

### 2.5. *γδ*T Cell Isolation and Purification

*γδ*T cells from mouse spleens were isolated and purified by the indirect magnetic bead separation method, using a PE selection kit and PE conjugated anti-mouse TCR *γ*/*δ* Clone: GL3 antibodies (BioLegend). Briefly, single-cell suspensions from mouse spleens were adjusted to 2*∗*10^8^/ml and transferred into flow cytometry tubes. Then, 10 *μ*l/ml mouse FcR blocker and 1 *μ*g/ml PE anti-mouse TCR *γ*/*δ* antibodies were added at room temperature for 15 minutes. Next, the PE selection cocktail was added at 100 *μ*l/ml to cells, at room temperature for 15 minutes. Magnetic nanoparticles were added at 50 *μ*l/ml to cells at room temperature for 10 minutes. Next, the mixture containing the cells was adjusted to a total volume of 2.5 mL with PBS containing 2% FBS and 1 mM EDTA. The tube was placed on a magnet for 5 minutes. Then, the magnet was washed 2–5 times, and cells in the tube were resuspended in RMPI 1640. For purification assessment, the cells were stained with anti-mouse CD3e PE-Cyanine5.5 for 30 minutes at 4°C in the dark and detected by flow cytometry.

### 2.6. Podocyte Apoptosis Assay

Forty-eight hours after LPS treatment with or without* γδ*T cells, podocytes were digested with 0.02% pancreatin (without EDTA) and collected. Podocyte apoptosis was assessed with Annexin-PI apoptosis detection kit (KeyGen BioTECH) according to the manufacturer's instructions.

### 2.7. Western Blotting

Kidney tissues were homogenized and lysed in RIPA lysis buffer (Bioteke Corporation, China). Cells were lysed in RIPA lysis buffer directly. After centrifugation, supernatants were collected and protein concentrations were detected using a BCA protein concentration detection kit (Beyotime, China). Equal amounts of protein were separated by 7.5–12.5% SDS-polyacrylamide gel electrophoresis (PAGE) (Beyotime, China) and transferred onto PVDF membranes. The membranes were blocked with 2% BSA for 1 h at room temperature and incubated with primary antibodies at 4°C overnight. The primary antibodies used were as follows: anti-SRC (phosphor Y529) antibodies (Abcam, 1 : 10000), mouse B7-1/CD80 affinity purified polyclonal antibodies (R&D system, 1 *μ*g/ml), and anti-GAPDH polyclonal antibodies (Proteintech, 1 : 20000). Blots were then washed and incubated with peroxidase-conjugated affinipure goat anti-rabbit or rabbit anti-goat secondary antibodies (IgG-HRP; ZSGB-BIO, Beijing, China, 1 : 5000) at room temperature for 1 h. After several washes, visualization was performed on an ECL Western blotting detection system (GE healthcare; RPN2106).

### 2.8. Quantitative Real Time Polymerase Chain Reaction (qRT-PCR)

Total RNA from mouse kidney and podocyte samples was extracted and purified with High Purified Total RNA Rapid Extraction Kit (BioTech Corporation, Beijing, China) according to the manufacturer's instructions. Reverse transcription was carried out with HiScript II Q Select RT SuperMix for qPCR (Vazyme) according to the manufacturer's instructions. Then, qPCR was performed using ChamQ SYBR qPCR Master Mix (Vazyme Code: Q311-02), in the following conditions: 30 seconds at 95°C, 40 cycles of denaturation at 95°C for 5 s, annealing at 60°C for 30 s, and extension at 72°C for 60 s. The primers were designed with the Primer Express software, as follows: B7-1 forward, 5′-TGTATGCCCAGGAAACAGGT-3′ and reverse 3′-AGCCCGAYCACCACTGATTA-5′; CCL2, forward 5′-GCTACAAGGATCACCAGCAG-3′ and reverse 3′-GTCTGGACCCATTCCTTCTTGG-5′; CCL5, forward 5′-CCTGCTGCTTTGCCTACCTCTC-3′ and reverse 3′-ACACACTTGGCGGTTCCTTGGA-5′; GAPDH, forward 5′-CATCACTGCCACCCAGAAGACTG-3′ and reverse 3′-ATGCCAGTGAGCTTCCCGTTCAG-5′. GAPDH was used as an endogenous reference gene. Data were analyzed using the 2-DDCt method and expressed as fold change in expression with respect to the control group (unstimulated cells).

### 2.9. Immunofluorescence

Fresh mouse kidney samples were stored in liquid nitrogen and sliced into 4 mm thick sections using a cryomicrotome. Frozen sections were stored at −80°C until use. Podocytes were fixed with 4% paraformaldehyde for 15 minutes and permeabilized with 0.1% TRITON X-100 in PBS. Nonspecific antigens were blocked by incubation with 2% BSA. For* γδ*T cell detection, direct immunofluorescence was used. Frozen sections were washed with PBS for three times (5 minutes each). Then, the frozen sections were covered and incubated with anti-TCR gamma and anti-TCR delta antibody (GL3) antibodies (FITC) (Abcam) overnight at 4°C in the dark. For B7-1/CD80, synaptopodin and phosphor-SRC detection, indirect immunofluorescence was used, with mouse B7-1/CD80 affinity purified polyclonal antibodies (R&D system, 10 *μ*g/ml), anti-mouse synaptopodin antibodies (Novus Biologicals, 1 : 50), and anti-mouse anti-SRC (phospho Y529) antibodies (Abcam, 1 : 100). Secondary antibodies were Alexa Fluor® 488 conjugated affinipure donkey anti-rabbit IgG (H + L) (Proteintech, 1 : 200), Alexa Fluor 555 conjugated affinipure donkey anti-goat IgG (H + L) (Proteintech, 1 : 200), and Alexa Fluor 555 conjugated affinipure donkey anti-rabbit IgG (H + L) (Proteintech, 1 : 200). For F-actin detection, Phalloidin-FITC (sigma, 50 ug/ml) labelled was purchased. Counterstaining was performed with DAPI staining solution (Beyotime, 1 : 20). Tissue and podocyte samples were observed by fluorescence microscopy. The experiments were repeated at least three times.

### 2.10. Statistical Analysis. 

Data are mean ± SEM. One-way analysis of variance (ANOVA) and *t*-test were used for group comparisons. *P* < 0.05 (*∗*) was considered statistically significant. All statistical analyses were performed with SPSS 13.0.

## 3. Results

### 3.1. LPS Induced Nephropathy Simulates MCD

**Figure 1:**
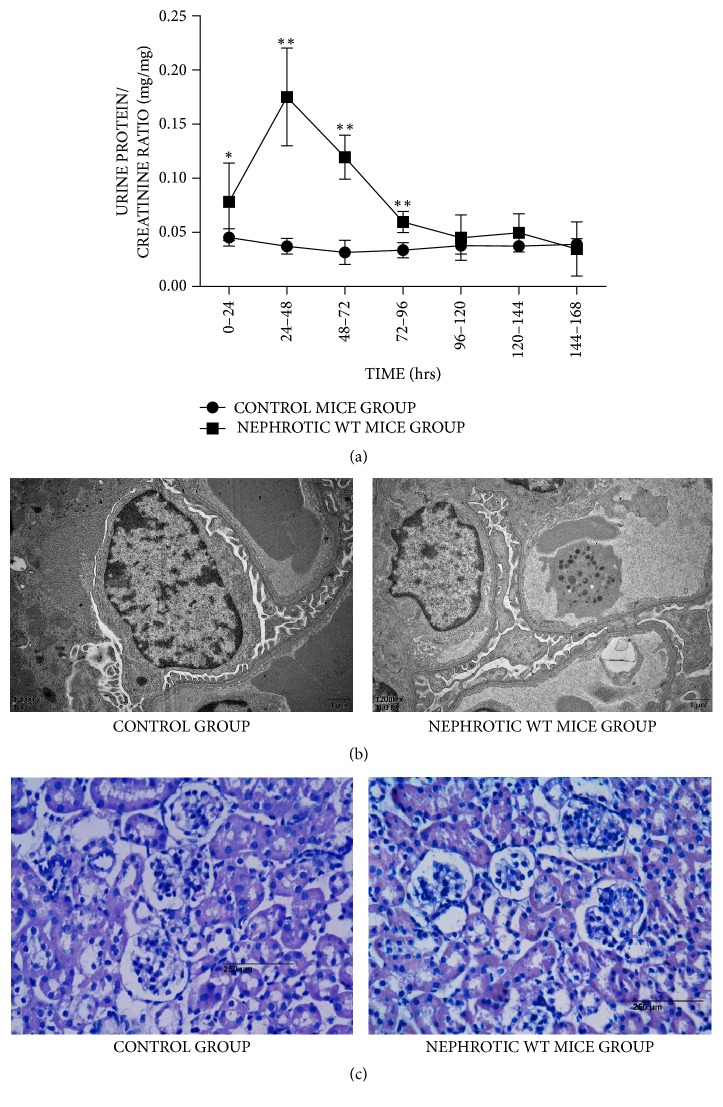
(a) WT mice were treated with LPS intraperitoneally (*n* = 7). Control group mice were treated with PBS. Then urine was collected before and at 0–24, 24–48, 48–72, 72–96, 96–120, 120–144, and 144–168 hours after treatment. The detection of urine protein/creatinine ratio showed that albuminuria of mice treated with LPS appeared as soon as 0–24 hours after treatment (^*∗*^*P* < 0.05) and then reached the peak after 24–48 hours (^*∗∗*^*P* < 0.01). The albuminuria declined from 48 hours to 96 hours and finally returned to normal. (b) Compared to control mice, significant foot process effacement was detected by electron microscope in WT mice treated with LPS. (c) Hematoxylin and eosin stain was used (original magnification: ×400). No obvious change was found in kidney tissues of WT mice treated with LPS.

After intraperitoneal injection of LPS, a transient protein elevation in WT mouse urine was observed. As shown in Figure 1(a), urine protein levels immediately increased at 0–24 hours after LPS injection (*n* = 7; 0.0454 ± 0.00807 for controls versus 0.0782 ± 0.0358 for LPS; ^*∗*^*P* < 0.05) and peaked at 24–48 hours (*n* = 7; 0.0372 ± 0.00720 and 0.175 ± 0.0451 for control and LPS groups, resp.; ^*∗∗*^*P* < 0.01). Then, urine protein levels decreased from 48 to 96 hours but still showed significant increase (*n* = 7; 48–72 hours, 0.0316 ± 0.0112 and 0.120 ± 0.0203 for control and LPS groups, resp., [*P* < 0.01]; 72–96 hours, 0.0336 ± 0.00697 and 0.0597 ± 0.00980 for control and LPS groups, resp., [*P* < 0.01]). After 96 hours, urine protein amounts returned to normal. Electron microscopy revealed that compared with control mice, WT mice showed kidneys with foot process effacement after LPS treatment (Figure 1(b)). However, light microscopy showed no glomerulosclerosis (Figure 1(c)). The findings indicated that LPS nephropathy simulates MCD.

### 3.2. LPS Nephropathy Induces *γδ*T Cell Infiltration, While *δ*TCR Knockout Attenuates LPS Nephropathy

**Figure 2:**
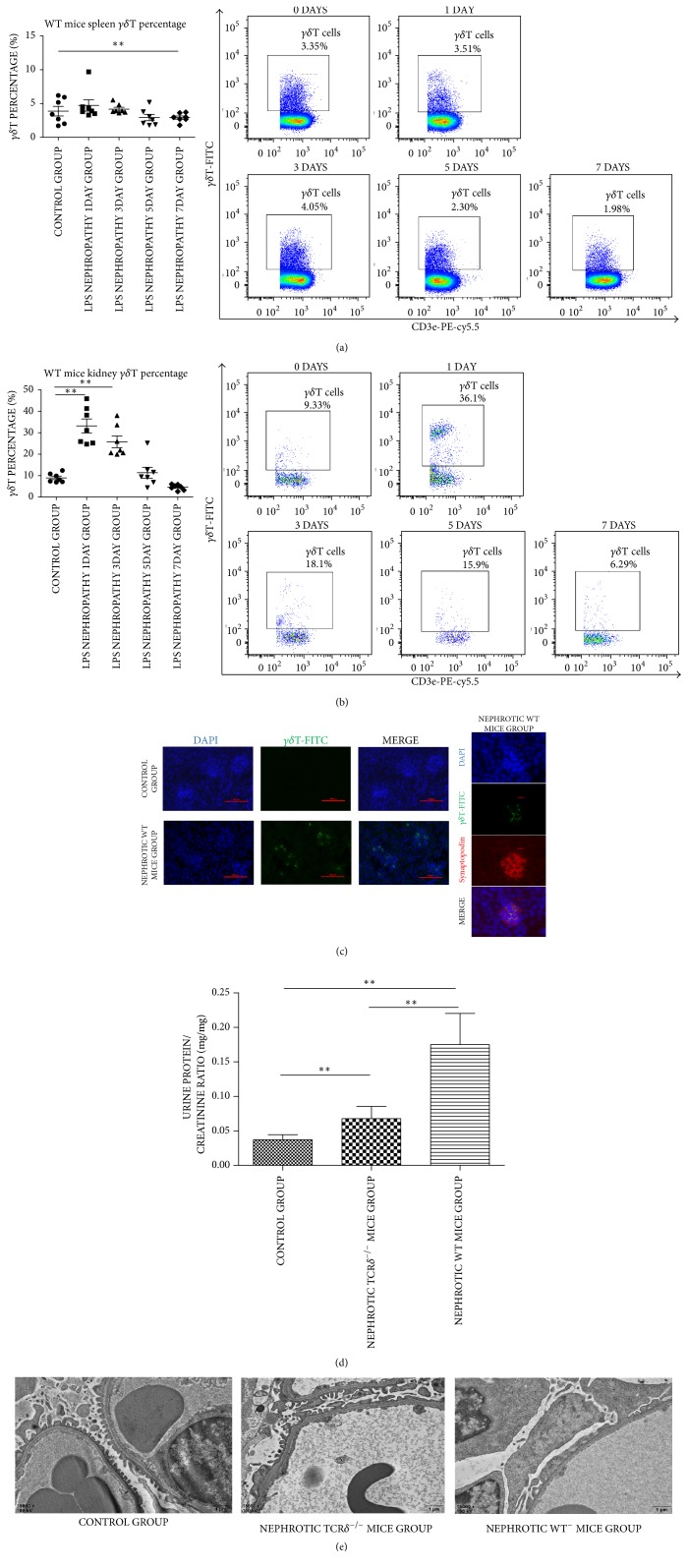
WT mice were treated with LPS, and *γδ*T cells in kidney and spleen were detected by flow cytometry at 0, 1, 3, 5, and 7 days after LPS treatment (*n* = 7). (a) In spleen, no significant difference was detected between control mice group and nephrotic WT mice group at 1st, 3rd, and 5th day (*P* > 0.05). However, compared to control group, *γδ*T cells percentage declined at the seventh day (^*∗∗*^*P* < 0.01). (b) In kidney, *γδ*T cells increased significantly at the first day (^*∗∗*^*P* < 0.01) and the third day (^*∗∗*^*P* < 0.01) and finally returned to normal at the fifth day (*P* > 0.05). (c) The *γδ*T cells were detected in kidney tissue by immunofluorescence. Moreover, colabelling result of *γδ*T cells and synaptopodin revealed *γδ*T cells deposited in glomeruli rather than other parts (*n* = 3). (d) At 24–48 hours after LPS treatment, urine protein/creatinine ratio of nephrotic TCR*δ*^−/−^ mice group (*n* = 5) was higher than control group (*n* = 7) (^*∗∗*^*P* < 0.01). However, it was significantly lower than that of nephrotic WT mice group (*n* = 7) (^*∗∗*^*P* < 0.01). (e) Electron microscope results revealed the degree of foot process effacement was alleviated in nephrotic TCR*δ*^−/−^ mice group than nephrotic WT mice group.

We detected* γδ*T cells in spleen and kidney samples from WT mice by flow cytometry at 0, 1, 3, 5, and 7 days after LPS treatment. A significant elevation of* γδ*T cells in kidney samples was observed immediately from the first day (8.94 ± 2.12, 33.2 ± 8.62, and 25.8 ± 7.24 for 0, 1, and 3 days, resp., [all *P* < 0.01]) and returned to normal at 5 days (11.4 ± 6.71; *P* > 0.05) (Figure 2(b)). Although no significant differences were found between spleen *γδ*T cells in control and LPS nephropathy group mice at 1st, 3rd, and 5th days, a significant reduction in *γδ*T cells was observed at the 7th day after LPS treatment, which could result from *γδ*T cells migration (control group: 3.87 ± 1.88, versus 7 days: 2.92 ± 0.64, ^*∗∗*^*P* < 0.01) (Figure 2(a)). The location of* γδ*T cells in mouse kidney samples was observed by immunofluorescence. The results of colabelling* γδ*T cells with synaptopodin revealed that* γδ*T cells were located in glomeruli rather than renal tubules (Figure 2(c)). These findings indicated that* γδ*T cells may play a vital role in pathological changes observed in the glomeruli. Furthermore, ten eight-week-old female TCR*δ*^−/−^ mice were injected with LPS, and two died within 24 hours; 3 were sacrificed at 24 hours after LPS treatment for electron microscopy. Urine from five mice was collected from 24 to 48 hours for protein quantitation. Urine protein levels were higher in nephrotic TCR*δ*^−/−^ mice than in the control group, but much significantly lower than in nephrotic WT mice (Figure 2(d)). In addition, electron microscope indicated that the degree of foot process effacement was alleviated in nephrotic TCR*δ*^−/−^ mice (Figure 2(e)). These results showed that* γδ*T cells could promote foot process injury in LPS induced nephropathy.

### 3.3. CD28 Loss in *γδ*T Cells and B7-1 Expression Are Induced in LPS Nephropathy

**Figure 3:**
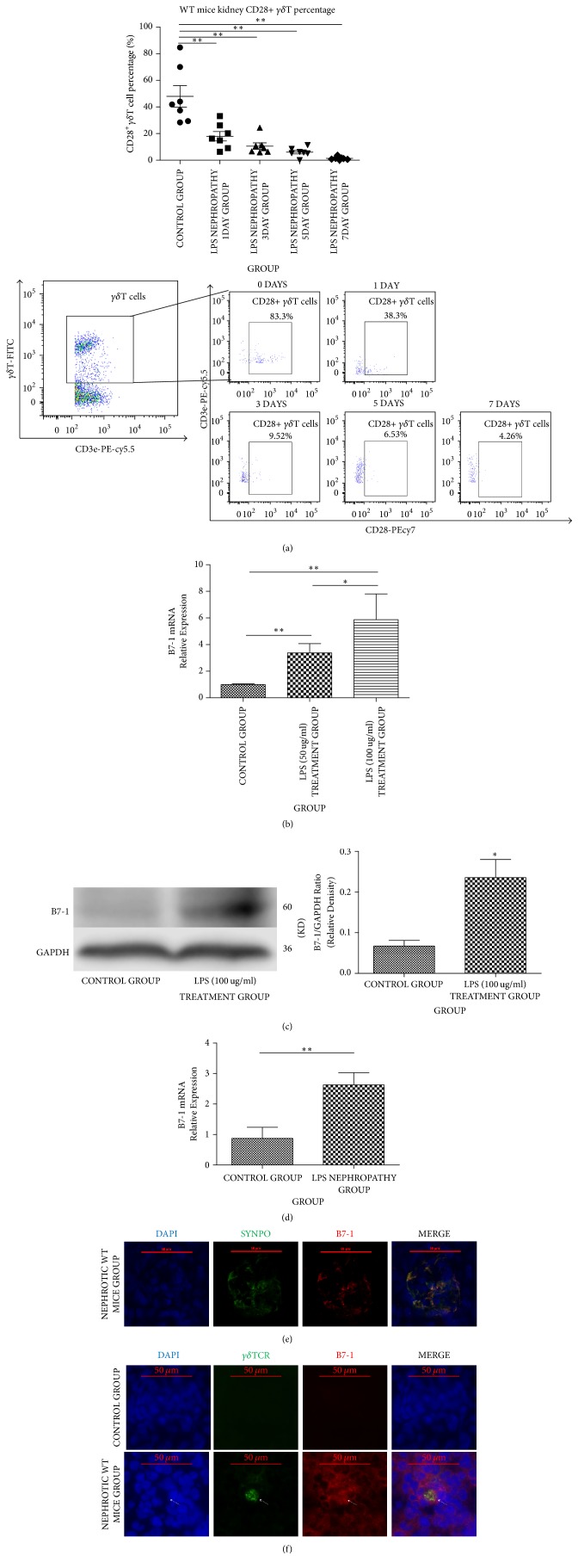
(a) WT mice were treated with LPS, and CD28^+^*γδ*T cells in kidney were detected by flow cytometry at 0, 1, 3, 5, and 7 days after LPS treatment (*n* = 7). CD28^+^*γδ*T cells decreased significantly from the first day (^*∗∗*^*P* < 0.01). (b) Podocytes were treated with LPS (50 ug/ml and 100 ug/ml); ^*∗∗*^*P* < 0.01 and ^*∗*^*P* < 0.05. Quantitative-RT-PCR detection result revealed that compared to control group, podocytes treated with LPS had higher B7-1 RNA expression and increased positively with LPS concentration. (c) Western blotting detection showed higher B7-1 expression in podocytes treated with LPS (100 ug/ml) than control group; ^*∗*^*P* < 0.05. (d) B7-1 gene expression in kidney tissue of nephrotic WT mice group was threefold higher than that of control group (*n* = 5, ^*∗∗*^*P* < 0.01). (e) Double labelling with synaptopodin (synpo) showed B7-1 expression was restricted to podocytes. (f) Immunofluorescence image showed B7-1 (red) and *γδ*T cells (green) were detected in glomeruli of nephrotic WT mice group. And their position was near and even overlapped. For experiments in vitro, all measurements were performed in duplicate.

We detected CD28^+^*γδ*T cells in kidney samples. Flow cytometry showed that although the total* γδ*T percentage was increased, CD28 expression on the cell membrane was significantly reduced (control, 48.0 ± 21.3; 1st day, 18.0 ± 9.39; 3rd day, 10.6 ± 6.46; 5th day, 6.05 ± 3.48; 7th day, 1.34 ± 1.18; all *P* < 0.01) (Figure 3(a)). CD28 downregulation is a marker of* γδ*T activation [2]. Next, RNA from mouse kidney tissue samples was extracted for B7-1 qRT-PCR detection. The results showed that B7-1 gene expression in nephrotic WT mice was threefold higher than that of the control group (2.25 ± 0.656 versus 0.723 ± 0.280, ^*∗∗*^*P* < 0.01) (Figure 3(d)). Podocytes were exposed to different doses of LPS (50 *μ*g/ml and 100 *μ*g/ml). Interestingly, B7-1 gene expression increased with LPS concentration (control, 0.9946 ± 0.06252; LPS 50 *μ*g/ml, 3.38 ± 0.693 [^*∗∗*^*P* < 0.01]; LPS 100 *μ*g/ml, 5.87 ± 1.93 [^*∗∗*^*P* < 0.01] (Figure 3(b)). Western blotting also showed increased B7-1 protein expression in podocytes treated with LPS (100 *μ*g/ml) (Figure 3(c)). Furthermore, double labelling with synaptopodin confirmed B7-1 expression was restricted to podocytes in kidney tissues (Figure 3(e)). Then, the spatial relation of B7-1 and* γδ*T cells was investigated with frozen mouse kidney tissue sections by immunofluorescence. As shown in Figure 3(f),* γδ*T cells were very close to and even overlapped with B7-1 positive areas in nephrotic WT mice, indicating the spatial relation between* γδ*T and B7-1. Taken together, the characteristics of CD28^+^*γδ*T cells and immunofluorescence data indicate that* γδ*T affected podocytes homeostasis by binding CD28 and B7-1, which could activate SRC kinase.

### 3.4. Phosphor-SRC Activation Is Increased in LPS Nephropathy and Podocyte Injury Is Exacerbated during Coculture with *γδ*T Cells

**Figure 4:**
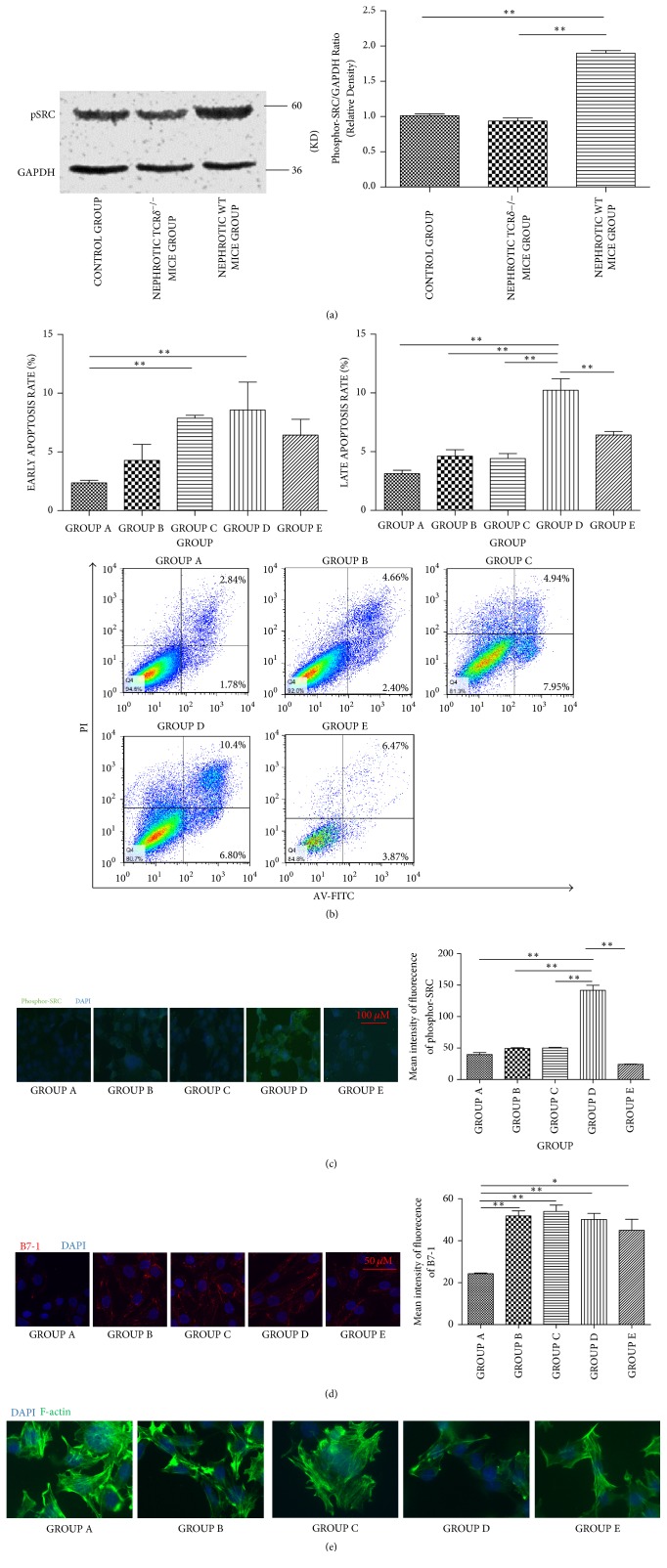
(a) Western blotting analysis indicated higher phosphor-SRC expression in nephrotic WT mice group than control group and nephrotic TCR*δ*^−/−^ mice group. (b) Podocytes apoptosis rate detection by Annexin V-PI apoptosis detection kit. Both of the early and late apoptosis rate were analyzed. Compared to control group, the early apoptosis rate of Groups C and D both significantly increased (^*∗*^*P* < 0.05). However, compared to other groups, only Group D had higher late apoptosis rate (^*∗*^*P* < 0.05). Group E, which was treated with CTLA4-Ig, could alleviate both the early and late apoptosis of Group D. ((c) and (d)) The phosphor-SRC and B7-1 of Podocytes was detected by cyto-immunofluorescence; ^*∗*^*P* < 0.05 and ^*∗∗*^*P* < 0.01. Phosphor-SRC expression was higher in Group D than other groups (142 ± 5.69 for Group D, compared with 39.7 ± 5.69, 49.3 ± 2.31, 50.0 ± 1.73, and 24.0 ± 1.00 for Groups A, B, C, and E, resp., all ^*∗∗*^*P* < 0.01). B7-1 expression was induced by LPS (24.3 ± 0.612, 51.8 ± 4.11, 53.9 ± 5.21, and 50.2 ± 4.97 for Groups A to D, resp., all ^*∗∗*^*P* < 0.01, and 45.0 ± 9.07 for Group E, compared to Group A as well, ^*∗*^*P* < 0.05); however, there was no significant difference of B7-1 expression in Groups B-E. (e) Immunofluorescence staining for F-actin (green).

Next, we assessed the changes of phosphor-SRC in mouse kidney. Phosphor-SRC was overtly activated in nephrotic WT mice compared with control and nephrotic TCR*δ*^−/−^ mice (Figure 4(a)). To further explore the underlying mechanism, the pathway was assessed in vitro. Mouse podocytes (MPCs) were divided into five groups as described in the experimental section. Groups B, C, D, and E were treated with 100 *μ*g/ml LPS. After 48 hours of stimulation, podocytes were used for apoptosis analysis and phosphor-SRC and B7-1 changes were observed by cyto-immunofluorescence. Early and late apoptosis rates were analyzed as well. As shown in Figure 4(b), compared with other groups, early apoptosis rates in Groups C and D were increased. However, only the late apoptosis rate in Group D was elevated significantly (Group D, 10.2 ± 1.95; versus, Group A, 3.14 ± 0.579; Group B, 4.62 ± 1.11; Group C, 4.42 ± 0.729; Group E, 6.43 ± 0.588; all ^*∗∗*^*P* < 0.01). The results of cyto-immunofluorescence showed that podocytes of Group D expressed stronger phosphor-SRC (immunofluorescence intensity: Group D, 142 ± 13.9; versus. Group A, 40.3 ± 5.68; Group B, 49.3 ± 2.30; Group C, 50.0 ± 1.73; Group E 24.0 ± 1.00, all ^*∗∗*^*P* < 0.01) (Figure 4(c)). Notably, Group E, which is treated with the CD28/B7-1 blocker CTLA4-Ig, had lower podocyte apoptosis rate and phosphor-SRC expression compared with Group D. The immunofluorescence of B7-1 revealed that, compared to Group A, B7-1 was induced by LPS in Group B-E and had no significant difference among Group B-E (immunofluorescence intensity: Group A, 24.3 ± 0.612; versus. Group B, 51.8 ± 4.11; Group C, 54.0 ± 5.21; Group D, 50.2 ± 4.97; and Group E, 45.0 ± 9.07, all *P* < 0.05) (Figure 4(d)). Phalloidin was used to detect F-actin of podocytes; the results displayed loss of stress fiber in Group D and recovered in Group E, indicating podocytes injury was exacerbated when the interaction of B7-1 and CD28 existed. These in vitro findings supported the notion that* γδ*T cells exacerbate podocyte injury via the CD28/B7-1-phosphor-SRC kinase pathway.

### 3.5. CCR2/CCL2 Induces *γδ*T Cell Migration

**Figure 5:**
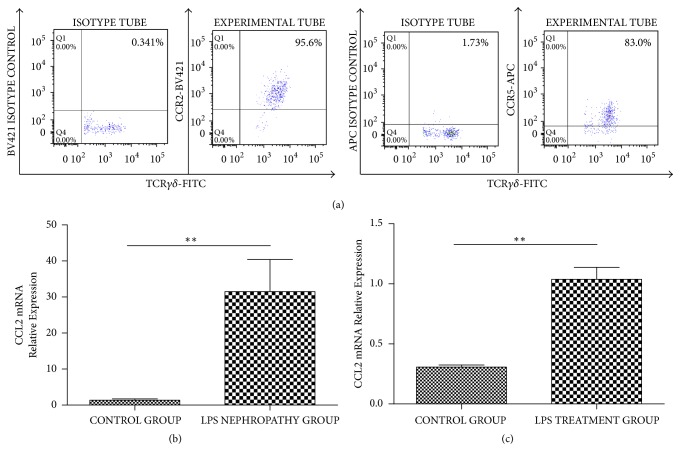
(a) CCR2^+^*γδ*T cells and CCR5^+^*γδ*T cells were detected by flow cytometry. CCR2^+^*γδ*T cells accounted for more than 95% of total *γδ*T cells in LPS nephropathy mice kidney. However, CCR5^+^*γδ*T cells accounted for only 70–80% of total *γδ*T cells. (b) CCL2 gene expression in WT mice LPS nephropathy group was significantly higher than in control mice (*n* = 4) (^*∗∗*^*P* < 0.01). (c) 48 hours after LPS treatment, podocytes had significantly higher CCL2 gene expression than control group (^*∗∗*^*P* < 0.01).

Finally, we assessed CCR2 and CCR5 expression on* γδ*T cells by flow cytometry. CCR2^+^*γδ*T cells accounted for more than 95% of all* γδ*T cells in nephrotic WT mouse kidney specimens. However, CCR5^+^*γδ*T cells accounted for only 70–80% of total* γδ*T cells (Figure 5(a)). Quantitative-RT PCR revealed increased CCL2 (CCR2 ligand) levels in the kidney tissue of nephrotic WT mice (control, 1.34 ± 0.763 versus. nephrotic WT mice, 31.5 ± 17.8, ^*∗*^*P* < 0.05) (Figure 5(b)) and podocytes treated with LPS (control, 0.307 ± 0.0467 versus. LPS treatment group, 1.04 ± 0.244, ^*∗∗*^*P* < 0.01) (Figure 5(c)) compared with respective control groups. These findings suggest CCR2 and CCL2 constitute the receptor and chemokine, inducing* γδ*T migration into the kidney.

## 4. Discussion

Podocyte injury is considered the most important pathological feature in MCD. As terminally differentiated cells, podocytes rarely recover from injury. Therefore, effective therapeutic methods should prevent progressive podocyte injury. However, the uncertain mechanism remains a barrier in developing efficient treatments for MCD. Based on previous findings, we assessed MCD from an immunological perspective. Consistent with other studies [16, 18], experimental outcomes such as transient proteinuria and foot process effacement indicated that LPS nephropathy in mice could be an appropriate animal model simulating human MCD.

As innate-like immune cells,* γδ*T cells are activated at the early stage of disease generation and development [19]. Studies demonstrated that* γδ*T cells could constitute the aggravating factor in several kidney diseases [6]. Previously, we found that* γδ*T amounts increase in peripheral blood from children with MCD [20]. Therefore, we hypothesized that* γδ*T cells could also be a risk factor in MCD. We firstly demonstrated infiltration of* γδ*T cells in kidneys from WT mice with LPS nephropathy by flow cytometry and immunofluorescence. Then, TCR*δ*^−/−^ mice were evaluated as well. Interestingly, compared with control group mice, TCR*δ*^−/−^ animals treated with LPS had slight proteinuria. However, proteinuria in TCR*δ*^−/−^ mice treated with LPS was much less severe than that of WT mice with LPS nephropathy. These preliminarily results supported our speculation.

B7-1, also termed as CD80, is considered a marker of immune cells. However, several studies have reported B7-1 expression on podocytes. B7-1 can be induced in LPS nephropathy and has been also detected in kidney tissue and urine samples from PNS patients [7, 8]. Consistent with these findings, we also demonstrated that B7-1 is barely expressed in control mice and podocytes without stimulation. However, Western blot, qRT-PCR, and immunofluorescence consistently showed high B7-1 expression in kidney tissue specimens from WT mice with LPS nephropathy and LPS treated podocytes. Double labelling detection of B7-1 and synaptopodin showed B7-1 was restricted to podocytes. Furthermore, the spatial relationship of* γδ*T cells and B7-1 was analyzed by immunofluorescence. Interestingly,* γδ*T cell infiltration was closed to and even overlapped with B7-1 positive areas. Then, we detected CD28 loss on* γδ*T cells by flow cytometry. CD28 is a B7-1 ligand. Its disappearance is also a marker for* γδ*T cell activation. Upon binding with B7-1, CD28 is suppressed. As shown above, CD28^+^*γδ*T cell percentage in the kidney of mice with LPS nephropathy was significantly lower than that of control mice, indicating that CD28 in* γδ*T cells had likely bound B7-1 on podocytes. However, whether* γδ*T cells affect podocyte homeostasis directly remains unclear. Therefore, more in vitro experiments are required.

For in vitro experiments, 0.4 *μ*m transwell plates were employed. After addition of* γδ*T cells to the upper chamber with podocytes in the lower chamber, the two cell types were separated. However, cytokines produced by* γδ*T cells could diffuse into the lower chamber. Podocytes were divided into five groups, including Groups A, B, C, D, and E, representing the control group, LPS treatment group without* γδ*T cells, LPS treatment group cultured with* γδ*T cells in the upper well of the transwell, LPS treatment group cocultured with* γδ*T cells, and LPS treatment group cocultured with* γδ*T cells and treated with CTLA4-Ig, respectively. Podocytes were exposed to LPS. After 48 hours of stimulation, the early apoptosis rates of Groups C and D were significantly higher compared with the control value. However, only Group D had higher late apoptosis rate. These findings indicated that despite the presence of both indirect (such as molecule) and direct interactions, direct interactions of* γδ*T cells and podocytes remain the main pathogenic pathway. Notably, the podocyte apoptosis rate of Group E was slightly higher compared with those of Groups A, B, and C, but significantly lower than that of Group D, suggesting CTLA4-Ig could prevent progressive injury in podocytes. CTLA4-Ig is a CD28/B7-1 blocker; it has high affinity for B7-1 and competes with CD28 for its binding. Since CD80 detection in podocytes, several studies have assessed the therapeutic effects of CTLA4-Ig in vivo and in vitro [21, 22]. Yu and colleagues revealed that Abatacept, a CTLA4-Ig drug, could alleviate disease progression in cases with B7-1 positive proteinuric kidney disease [12]. However, the underlying mechanism remains unidentified. The current results suggested that CTLA4-Ig protects podocytes mainly by blocking CD28 binding to and B7-1.

As shown above, phosphor-SRC expression was much higher in Group D and returned to baseline when CTLA4-Ig was added which can block the interaction between CD28 and B7-1. However, the B7-1 expression was induced by LPS treatment and had no significant difference among Groups B-E. The results indicated phosphor-SRC was induced by the interaction between* γδ*T cells and B7-1 rather than B7-1 itself. SRC is a nonreceptor tyrosine kinase that affects phosphorylation or dephosphorylation of diverse signaling proteins. It participates in multiple tyrosine signaling pathways, including cytoskeletal dynamics. Buvall and colleagues reported that phosphor-SRC activation could lead to synaptopodin dephosphorylation, promoting synaptopodin binding to the serine/threonine phosphatase calcineurin. This leads to loss of 14–3-3 binding, resulting in synaptopodin degradation and loss of stress fibers ultimately [13]. As shown above, podocytes cocultured with* γδ*T cells had higher apoptosis rate and phosphor-SRC expression levels compared with other groups. Moreover, the F-actin detection of podocytes showed F-actin loss in Group D. However, phosphor-SRC detection in Group E revealed that CD28/B7-1 blockage through CTLA4-Ig could inhibit phosphor-SRC activation and progressive injury in podocytes. These results demonstrated that direct interactions between* γδ*T cells and podocytes may activate intracellular phosphor-SRC, leading to podocyte injury.

In conclusion,* γδ*T cells exacerbate podocyte injury via binding to B7-1 with further phosphor-SRC activation, leading to altered cytoskeleton. CTLA4-Ig, a CD28/B7-1 blocker, could prevent progressive injury in podocytes, representing a potential therapeutic tool for pediatric PNS.

In addition, we assessed chemokines and their receptors in LPS nephropathy. Compared with control mice, WT mice with LPS nephropathy had higher CCL2 gene expression in the kidney. Meanwhile, CCR2+* γδ*T cells accounted for more than 95% of all* γδ*T cells that infiltrated the kidney. Therefore,* γδ*T cell migration was mainly induced by the CCR2/CCL2 axis.

We found that the interaction of CD28 from* γδ*T cells and B7-1 from podocytes induces phosphor-SRC activation, leading to podocyte injury. Blockage of such interaction, for example, by incubation with CTLA4-Ig, may be a desirable treatment, especially in children with PNS who are not sensitive to corticosteroids.
